# The 2006 California Heat Wave: Impacts on Hospitalizations and Emergency Department Visits

**DOI:** 10.1289/ehp.11594

**Published:** 2008-08-22

**Authors:** Kim Knowlton, Miriam Rotkin-Ellman, Galatea King, Helene G. Margolis, Daniel Smith, Gina Solomon, Roger Trent, Paul English

**Affiliations:** 1 Health and Environment Program, Natural Resources Defense Council, New York, New York, USA; 2 Environmental Health Sciences Department, Mailman School of Public Health, Columbia University, New York, New York, USA; 3 Health and Environment Program, Natural Resources Defense Council, San Francisco, California, USA; 4 California Department of Public Health, Environmental Health Investigations Branch, Richmond, California, USA; 5 Department of Internal Medicine, School of Medicine, University of California Davis, Sacramento, California, USA; 6 Pediatric Environmental Health Specialty Unit, California Poison Control System San Francisco, University of California San Francisco, San Francisco, California, USA; 7 School of Medicine, University of California San Francisco, San Francisco, California, USA; 8 California Department of Public Health, Epidemiology and Prevention for Injury Control Branch, Sacramento, California, USA

**Keywords:** climate change, electrolyte imbalance, emergency department, global warming, heat stroke, heat wave, hospitalization, morbidity, renal failure, temperature

## Abstract

**Background:**

Climate models project that heat waves will increase in frequency and severity. Despite many studies of mortality from heat waves, few studies have examined morbidity.

**Objectives:**

In this study we investigated whether any age or race/ethnicity groups experienced increased hospitalizations and emergency department (ED) visits overall or for selected illnesses during the 2006 California heat wave.

**Methods:**

We aggregated county-level hospitalizations and ED visits for all causes and for 10 cause groups into six geographic regions of California. We calculated excess morbidity and rate ratios (RRs) during the heat wave (15 July to 1 August 2006) and compared these data with those of a reference period (8–14 July and 12–22 August 2006).

**Results:**

During the heat wave, 16,166 excess ED visits and 1,182 excess hospitalizations occurred statewide. ED visits for heat-related causes increased across the state [RR = 6.30; 95% confidence interval (CI), 5.67–7.01], especially in the Central Coast region, which includes San Francisco. Children (0–4 years of age) and the elderly (≥ 65 years of age) were at greatest risk. ED visits also showed significant increases for acute renal failure, cardiovascular diseases, diabetes, electrolyte imbalance, and nephritis. We observed significantly elevated RRs for hospitalizations for heat-related illnesses (RR = 10.15; 95% CI, 7.79–13.43), acute renal failure, electrolyte imbalance, and nephritis.

**Conclusions:**

The 2006 California heat wave had a substantial effect on morbidity, including regions with relatively modest temperatures. This suggests that population acclimatization and adaptive capacity influenced risk. By better understanding these impacts and population vulnerabilities, local communities can improve heat wave preparedness to cope with a globally warming future.

Deaths from heat waves in the United States and Europe have been described in a number of studies (Basu and Samet 2002; Donoghue et al. 2003; Hajat et al. 2006; Kinney et al. 2008; Medina-Ramon et al. 2006; Naughton et al. 2002; Semenza et al. 1996; Vandentorren et al. 2004), but there have been relatively few studies of heat-related morbidity (Kovats et al. 2004; Mastrangelo et al. 2006; Nitschke et al. 2007; Semenza et al. 1999). Increased mortality during heat waves has been attributed mainly to cardiovascular illness (13–90%) and diseases of the cerebrovascular (6–52%) and respiratory systems (up to 14%) (Kilbourne 1999), especially among the elderly (Kovats and Ebi 2006). Heat stress can rapidly become life threatening, especially among those with limited access to immediate medical attention (Mastrangelo et al. 2006). People with severe heat stroke symptoms have little time to seek treatment in emergency departments (EDs) or hospitals (Kovats and Ebi 2006; Kovats et al. 2004; Naughton et al. 2002; Semenza et al. 1996).

Previous studies have reported different patterns of morbidity in contrast to mortality patterns during heat waves. Hospital admissions during heat waves have been reported to increase among both older and younger adults, especially among adults living in institutions or engaging in outdoor activities involving exertion (Johnson et al. 2005; Kovats and Ebi 2006). Most admissions were for heat-related conditions, including heat exhaustion and heat stroke, dehydration and electrolyte disorders, and acute renal failure (Kovats and Ebi 2006; Mastrangelo et al. 2006; Semenza 1999; Semenza et al. 1999). Some increases in admissions for neurologic conditions and mental illnesses (Kovats and Ebi 2006) and ambulance transport for violence-related causes (Nitschke et al. 2007) have been reported. A study of emergency hospital admissions in London (Kovats et al. 2004) failed to find statistically significant increases in total emergency hospital admissions during extreme heat, although increases were found for respiratory and renal illnesses in children 0–4 years of age and for respiratory illness in people ≥ 75 years of age. In other studies, hospitalizations for cardiovascular diseases have decreased slightly (3%) during heat waves (Mastrangelo et al. 2006). The relationship between heat and morbidity in any specific area may be affected by local population demographics, economic well-being, underlying disease risk, the presence of vulnerable subpopulations, weather variability, physiologic acclimatization, and locally available adaptations (Kinney et al. 2008).

During mid- to late July 2006, an extreme heat wave affected much of the state of California, breaking daily maximum temperature records at seven sites and exceeding minimum temperature records at 11 meteorologic stations, especially from 16 through 26 July. Both the intensity and duration of the heat wave were exceptional, and records for most consecutive days reaching ≥ 100°F were broken in the Central Valley region (Kozlowski and Edwards 2007). The statewide mean temperature for July 2006 was 4°F above the long-term climatologic average, with some stations in central and southern California recording the warmest July on record (Edwards 2006). County coroners and medical examiners reported at least 140 deaths from extreme heat recorded between 15 July and 1 August 2006 (California Department of Health Services 2007).

This event offered a unique opportunity to study heat-related morbidity patterns in a large U.S. study area (> 163,000 square miles) and population (37 million California residents). Better understanding of the patterns of morbidity during heat waves is an important tool for public health practitioners, because more intense, more frequent, and longer duration heat waves are projected for the coming decades (Meehl and Tebaldi 2004). The health impacts of climate change are gaining considerable attention (Frumkin et al. 2008), with increases in heat wave–related illness and death among the most likely related challenges to public health (Confalonieri et al. 2007; Jackson and Naumoff Shields 2008; Kovats and Hajat 2008). The 2006 heat wave may thus represent a sentinel event of a major public health challenge associated with climate change. To evaluate the effects of extreme heat on morbidity during the 2006 heat wave, the California Department of Public Health and the Natural Resources Defense Council investigated hospitalizations and ED visits for certain illnesses to discern regional patterns by diagnosis, age, and race/ethnicity. One of the goals of the present study was to learn which illnesses are exacerbated by heat waves in California and lead people to seek medical attention, thus providing opportunities for early intervention and public education to prevent heat-related illness and death.

## Materials and Methods

### Health outcome data

The California Office of Statewide Health Planning and Development (Sacramento, CA) provided patient discharge data and ED visit files. Diagnoses in both sources are coded according to the *International Classification of Diseases, 9th Revision, Clinical Modification* (ICD-9-CM; Centers for Disease Control and Prevention 1979). Of the hospital admissions during this period, 41% occurred among those people who had been admitted from an initial ED visit. The ED data, on the other hand, included all ED visits, regardless of whether patients were subsequently admitted to the hospital (of which 16% were). Thus, our resulting data sets for all incidents of ED visits and inpatient hospitalizations were not mutually exclusive.

Heat waves exacerbate a wide range of preexisting illnesses, and excess morbidity and mortality associated with heat waves go beyond that classified formally as “heat related.” With this in mind, we classified morbidity outcome data into categories consistent with the major groups applied in previous heat wave hospitalization studies (notably Semenza et al. 1999) and included all diagnoses combined, all internal causes (ICD-9-CM codes 001–799.9), diabetes mellitus (250), disorders of fluid and electrolyte balance (276), cardiovascular diseases (390–398, 402, 404–429, 440–448), acute myocardial infarction (MI; 410), cerebrovascular diseases (430–438), respiratory illnesses (460–519), nephritis and nephrotic syndrome and nephrosis (580–589), acute renal failure (584), and heat-related effects (992).

Because risk appears to be greatest in very young and old persons, we used three age categories in the analysis: 0–4 years, 5–64 years, and ≥ 65 years of age. We used several race and ethnicity categories that have been previously applied in health vulnerability analyses in California: Asian/Pacific Islander, African American, Latino/Hispanic, Native American/Alaska Native, other, unreported race/ethnicity, and non-Hispanic white.

Some studies of heat wave–related hospitalizations have found that using primary discharge diagnoses alone can underestimate increases in some admissions (Kilbourne 1999; Semenza et al. 1999). To calculate rate ratios (RRs) among ED visits and hospitalizations, we combined the primary and the first nine secondary diagnoses listed in the discharge record; for example, we classified admissions that included a nephritis code, regardless of whether primary or secondary, as nephritis in this analysis. Because there may be appreciable variation in the order of the various cause codes in the primary (the condition that prompted the admission or visit, not necessarily the most severe condition) and secondary diagnoses, we combined the primary and the first nine secondary codes to lend more consistency to the heat-wave versus non-heat-wave descriptive epidemiology.

To estimate the number of excess hospital admissions and ED visits during the heat wave and describe the principal disease process treated, we evaluated primary discharge diagnoses separately to tabulate changes in the total numbers of individuals seeking treatment.

### Study area

**Figure 1:**
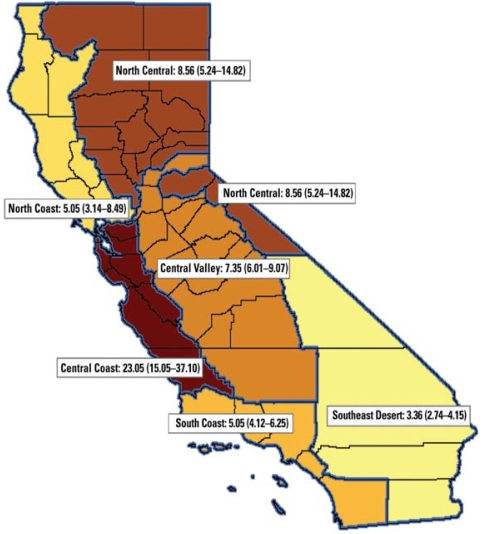
Map showing RRs (95% CIs) for ED visits for heat-related illnesses (ICD-9 code 992) among all ages, during the 15 July to 1 August 2006 heat wave, compared with a reference period (8–14 July and 12–22 August 2006). For the heat-wave morbidity analysis, we grouped counties into six regions, adapted from the U.S. Climate Divisions for California: Central Coast region (Alameda, Contra Costa, Monterey, San Benito, San Francisco, San Luis Obispo, San Mateo, Santa Clara, Santa Cruz Counties); Central Valley region (Amador, Calaveras, Fresno, Kern, Kings, Madera, Mariposa, Merced, Placer, Sacramento, San Joaquin, Stanislaus, Tulare, Tuolumne Counties); North Central region (Alpine, Butte, Colusa, El Dorado, Glenn, Lassen, Modoc, Mono, Nevada, Plumas, Shasta, Sierra, Siskiyou, Sutter, Tehama, Yolo, Yuba Counties); North Coast region (Del Norte, Humboldt, Lake, Marin, Mendocino, Napa, Solano, Sonoma, Trinity Counties); South Coast region (Los Angeles, Orange, San Diego, Santa Barbara, Ventura Counties); and Southeast Desert/Inland Empire region (Imperial, Inyo, Riverside, San Bernardino Counties).

For confidentiality, individual cell counts of five incidents or less were suppressed. This resulted in a high frequency of suppressed data in more sparsely populated counties. To avoid these issues in describing patterns of morbidity, we grouped California’s 58 counties into regions based on climate and population distribution, shown in Figure 1: Central Coast, Central Valley, North Central, North Coast, South Coast, and Southeast Desert/Inland Empire. These six regions, which we adapted from the U.S. Climate Divisions for California (Earth System Research Laboratory 2008), describe more-or-less contiguous county groups except in the case of the Central Valley region, which included Placer County, because most of its population resides in the western portion of the county.

### Definition of heat wave period

The definition of a “heat wave” varies by location, and different studies have applied various temperature metrics (Kinney et al. 2008). The variety of climate regimes across California would have required use of a variety of locally varying definitions of “heat wave.” Climatologic analysis of the 2006 California heat wave by meteorologic researchers suggested that, statewide, the most intense heat occurred from 16 through 26 July (Kozlowski and Edwards 2007). We chose to somewhat broaden the heat wave period in our analysis to the 18 days from 15 July through 1 August 2006 inclusive. This period encompasses the dates of the first and last reported heat-related deaths in California associated with the 2006 heat wave [California Department of Public Health (CDPH) 2008] and also better captures the onset of the event in many counties as well as the potential morbidity effects in the days immediately after the most extreme of the high-heat days.

Because ED data were available only for 2006, we selected a reference period in 2006. To minimize potential time-varying confounding effects, we selected a near-term summer reference period of the same duration (18 days) and with the same distribution of days of the week for the analysis: 8–14 July 2006 and 12–22 August 2006. Some previous heat mortality studies have found that the number of deaths caused by heat waves is partially offset by a temporary reduction in death rates in subsequent weeks (Braga et al. 2001; Huynen et al. 2001). It is uncertain whether similar morbidity displacement effects occur; for this reason the reference period did not include the days immediately after the heat wave. The reference period was more representative of normal temperature conditions for California (CalClim 2006).

### Statistical methods

Assuming that the California population changed little over the course of one summer, and selecting the reference period with the same number of days and distribution of days of the week as the heat wave period, the person-time units in the denominators of the two rates were equivalent. This allowed us to compare the ratio of the numbers of cases in the two time periods as an RR (Rothman and Greenland 1998). We calculated excess cases as the difference in the numbers of cases in the two periods. We calculated exact 95% confidence intervals (95% CIs) for the RRs using SAS version 9.1 (Daly 1992; SAS Institute, Inc., Cary, NC). We calculated RRs reported to two decimal places and 95% CIs for each of the six California regions and for the state as a whole across a range of cause–age and cause–race/ethnicity categories. We tested variation in the RRs across regions with a chi-square test for homogeneity (Rothman and Greenland 1998).

## Results

### Overall Morbidity During the 2006 California Heat Wave

In the 2006 California heat wave, we found dramatic increases across a wide range of morbidities statewide, with excess ED visits far outpacing excess hospitalizations. We found 501,951 ED visits during the 15 July to 1 August 2006 heat wave period compared with 485,785 visits in the non-heat-wave reference period. During the heat wave period, the excess number of ED visits was 16,166.

When we considered primary diagnosis plus the first nine underlying comorbidities in computation of RRs by region (Table 1), we found significant increases in the rates of all-cause, all-age ED visits in five of the six California regions (all except the Southeast Desert), ranging from RR = 1.03 (95% CI, 1.02–1.03) in the South Coast region to RR = 1.05 in the Central Coast (95% CI, 1.04–1.06) and North Coast (95% CI, 1.03–1.07) regions. Both all-cause and heat-related causes showed statistically significant variation across the six regions. The two regions that contributed the most to the test statistic (i.e., the regions that were the most discrepant from the overall mean) were the Central Coast (which had a higher RR than the average) and Southeast Desert (which had a lower than average RR) regions. We observed significantly increased rates of ED visits among each of the three age groups (0–4, 5–64, ≥ 65 years of age). We found significant increases in the rate of ED visits for most of the race/ethnicity groups statewide except for Native American/Alaska Natives and Asian/Pacific Islanders. Significant ED RRs for all causes and ages combined ranged from RR = 1.02 (95% CI, 1.01–1.03) for the African American race/ethnicity group up to RR = 1.04 (95% CI, 1.03–1.05) for the Latino/Hispanic group. Statewide, we found a significant increase in the rate of ED visits for all causes (RR = 1.03; 95% CI, 1.02–1.04). Heat-related illnesses, electrolyte imbalance, acute renal failure, nephritis and nephrotic syndrome, diabetes, and cardiovascular diseases were the main reasons for patients’ ED visits (Table 2).

We found 193,008 hospital admissions during the 2006 heat wave period compared with 191,826 admissions in the non-heat-wave 2006 reference period. During the heat wave defined for this study, the excess number of hospitalizations was 1,182. In contrast to the ED findings, the statewide rate of hospitalizations for all causes and all ages did not significantly increase (RR = 1.01; 95% CI, 1.00–1.01) (Table 2). During the heat-wave period, the main causes contributing to the excess hospitalizations included electrolyte imbalance, acute renal failure, nephritis and nephrotic syndrome, and heat-related illnesses (Table 2). None of the regions showed significantly elevated rates of hospitalizations (for all causes, all ages, all races/ethnicities combined) during the heat wave. We found no significantly increased rates of hospitalization in the three age groups. Considering possible race/ethnicity vulnerabilities from the statewide hospital data for all ages combined, the RRs were not significantly increased (Table 1).

### Internal Causes

The internal causes category represents all-cause morbidity minus injuries and poisonings. Regionally, we found increased rates of ED visits in five of the six individual regions (all except the Southeast Desert region), with RRs ranging from 1.02 (95% CI, 1.02–1.03) in the South Coast region to RR = 1.06 (95% CI, 1.05–1.07) in the Central Coast region (data not shown). We found significantly increased rates of internal-cause ED visits for each of the three age groups and for all race/ethnicity groups statewide (with the exception of Native American/Alaska Native) ranging from RR = 1.02 (95% CI, 1.01–1.04) for African Americans to RR = 1.04 (95% CI, 1.03, 1.05) for the Latino/Hispanic group. Statewide we found significantly elevated rates of ED visits (RR = 1.03; 95% CI, 1.03–1.04) for internal causes but not for hospitalizations (RR = 1.01; 95% CI, 1.00–1.01) (Table 2). None of the regions had significant increases in all-age internal cause hospitalizations, nor did any of the three age groups statewide.

### Specific Causes

#### Heat-related illness, electrolyte imbalance, acute renal failure, and nephritis/nephrotic syndrome

To take a closer look at the increased morbidity experienced during the heat wave, we evaluated patterns in the rate of ED visits and hospitalizations for 10 different causes across different geographical regions, age categories, and race/ethnicity groupings. The most striking findings were significantly elevated RRs in both ED visits and hospitalizations for heat-related illness, electrolyte imbalance, acute renal failure, and nephritis and nephrotic syndrome. We found elevated rates among the various geographic regions, age groups, and race/ethnicity categories, as shown in Table 3. In particular, the magnitude of the effects on heat-related illnesses was dramatic. Statewide, we found a more than 6-fold increase in heat-related ED visits (RR = 6.30; 95% CI, 5.67–7.01) and a more than 10-fold increase in heat-related hospitalizations (RR = 10.15; 95% CI, 7.79–13.43). We found even larger effects for specific geographic region, age, and race/ethnicity categories (Table 3).

#### Acute MI, cardiovascular diseases, cerebrovascular disease, diabetes, and respiratory illnesses

##### Geographic region

We found elevated rates of ED visits for all ages in the Central Coast region for diabetes (RR = 1.08; 95% CI, 1.04, 1.12), cardiovascular diseases (RR = 1.05; 95% CI, 1.02–1.09), and respiratory illnesses (RR = 1.05; 95% CI, 1.02–1.07). In contrast, we found no significant increases in hospitalizations for these causes by region.

##### Age

We found elevated rates of ED visits in the ≥ 65 years age group statewide for diabetes (RR = 1.04; 95% CI, 1.02–1.06) and respiratory illnesses (RR = 1.04; 95% CI, 1.02–1.06). In contrast, we found no significant increases statewide in hospitalizations for these causes in any of the age categories.

##### Race/ethnicity

We found elevated rates of ED visits among the white race/ethnicity group for diabetes (RR = 1.04; 95% CI, 1.02–1.06). Among the Latino/Hispanic race/ethnicity group, rates of acute MI (RR = 1.05; 95% CI, 1.01–1.08) and cardiovascular diseases (RR = 1.17; 95% CI, 1.02–1.34) were elevated for ED visits. Hospitalizations among this group were also elevated for cardiovascular diseases (RR = 1.04; 95% CI, 1.01–1.07). We found significantly increased hospitalization rates for respiratory illnesses statewide among Asian/Pacific Islanders (RR = 1.07; 95% CI, 1.02–1.14). We found no significant increases in RRs among African Americans for hospitalizations or ED visits in these other causes.

## Discussion

The July 2006 California heat wave had a substantial impact on morbidity throughout California, with variations across regions, race/ethnicity, and age subgroups. Many of the RR effect estimates were relatively small, but because the statewide heat wave exposed a very large population, these effects translate to a significant public health burden of what was largely preventable illness. One strength of the present study is that it offers information about the short-term increases in patient demand and the range of illnesses that may arise during heat waves as well as showing substantial regional variability.

The estimated number of excess ED visits (16,166) was far greater than the number of excess hospitalizations (1,182). In general, there are nearly three times more ED visits than hospitalizations in California (Office of Statewide Health Planning and Development 2007). Numerous factors influence whether a patient visiting an ED is subsequently admitted, among them individual hospital practices, patient insurance status, and the particular demographics and comorbidities of the individual. Although typically considered an indicator of a less severe level of morbidity than hospitalization, each ED visit represents a potentially serious health outcome that places demands on the health care system and clinicians. The dramatic effect of the 2006 heat wave on ED visits suggests that advance preparedness efforts should be undertaken to allow rapid adaptability when extreme weather events occur. These findings also suggest that including ED visits in describing the epidemiology of heat morbidity yields valuable information that a hospitalization-only study cannot.

The regional descriptive epidemiology provides insight into patterns of heat wave morbidity across climate zones. The Central Coast region (a typically cooler climate zone) contributed far more to the overall excess ED visits (28%) and to excess hospitalizations (47%) than would be expected based on overall state population (18%). This pattern suggests an important role for acclimatization and for factors related to the built environment. In San Francisco, for example, housing stock is less likely to have central air conditioning both because of its age and because of the cooler climate.

We found a far wider range of causes with significantly increased RRs for ED visits than for hospitalizations, and the ED patient population was younger than the population hospitalized during the heat wave. People ≥ 65 years of age comprised 52% of the excess hospitalizations but only 15% of excess ED visits (even though they comprised just 11% of the state’s population).

Including the ED visits as a parallel analysis with the hospitalizations enabled us to discern the adverse effects of heat on morbidity in the youngest (0–4 years) age group. We found that children statewide showed an increase in ED visits for electrolyte imbalance. This suggests that stronger messaging to parents, other caregivers, and school personnel to closely watch hydration for children during hot weather is a critical intervention to avoid excess pediatric ED visits during heat waves. Other significantly elevated ED RRs among California’s children included heat-related and internal causes. Thus, in addition to recognized heat vulnerabilities among those ≥ 65 years of age, preventive strategies to protect the health of the very young during heat waves should be devised.

The data suggest that both heat-related and electrolyte imbalance morbidities affected a wide range of race/ethnicity categories in the state. Data suppression and underreporting issues limited our ability to determine geographic variations in patient demographics relative to race/ethnicity, and the findings of this study suggest potential vulnerabilities related to race and specific illnesses that merit further investigation. For example, we found significantly increased rates of ED visits and hospitalizations for cardiac-related illnesses statewide (acute MI and cardiovascular disease cause codes) only among Latinos/Hispanics, which could be related to occupational heat exposures among Latino/Hispanic crop workers (Luginbuhl et al. 2008). In another example, we found significant increases in the rates of ED visits and hospitalizations among African Americans for multiple cause categories, including heat-related, electrolyte imbalance, acute renal failure, internal causes (ED only), and nephritis (hospitalizations only). Asian/Pacific Islanders had strongly elevated RRs for heat-related ED visits statewide (RR = 11.38; 95% CI, 5.53–27.14) but, unlike other major racial/ethnic groups, did not have significantly increased rates of either ED visits or hospitalizations for acute renal failure. Among the likely influences on this result are differing degrees to which EDs may be used for primary health care among different race/ethnicity groups. Although socioeconomic factors are important in determining heat vulnerability, that information for individual cases could not be evaluated in this study. However, newly released research (CDPH 2008) describes California county-level variations in socioeconomic and demographic determinants of heat vulnerability and represents an important companion to this work.

One limitation of this study is the issue of potential confounding or effect modification by air pollution, which could in particular affect cardiovascular and respiratory morbidities. Previous studies have produced conflicting results regarding pollution effects on heatrelated premature mortality (Basu and Samet 2002; Basu et al. 2005; Filleul et al. 2006; Hales et al. 2000; O’Neill et al. 2005a; Rainham and SmoyerTomic 2003; Ren et al. 2008). Although failure to control for air pollution can overestimate temperature–mortality associations, most studies have found that heatmortality effects persist even after adjustment for ozone and particulate matter. Recent research suggests that temperature effects on nonaccidental mortality in nine California counties from 1999 through 2003 were independent of air pollutants, including ozone, fine particulate matter, carbon monoxide, and nitrogen dioxide, none of which were significant confounders or effect modifiers (Basu et al. 2008). However, where ozone and temperature are highly correlated, some of the effects attributed to temperature could independently be attributable to ozone. Studies have yet to quantify the independent or interactive effects of ozone and heat on morbidity. This is an important topic for future research, which may employ study designs such as time-series or case–crossover analyses to evaluate the relative effects of temperature extremes versus air pollution upon morbidity in local populations or to examine the possible effects of lags in exposures (Kinney et al. 2008). This study focused on the aggregate effects of an 18-day heat wave period upon population morbidity across large geographic regions, so time-series methods were not appropriate.

The choice of reference period in heat wave analyses can affect the results, and we performed a sensitivity analysis to explore the impacts of applying an alternate reference period. The sensitivity test calculated RRs (and 95% CIs) for an 11-day heat wave period (16–26 July) encompassing the period of the most extreme heat, and a corresponding 11-day reference period (8–14 July and 13–16 August), selected to encompass the same distribution of week days as the heat wave, avoid the 4 July holiday period, and avoid seasonal and interannual variations in morbidity rates and population demographics. We calculated the effects of the shorter heat wave and reference period upon the number of statistically significant RRs across the range of possible hospitalization and ED visit age–cause categories in the state and the six regions. We found very little difference, relative to the main analysis’ results, for ED visits (4% decrease in the number of statistically significant age-cause categories). We found larger differences in the hospitalizations (25% increase in the number of statistically significant age–cause categories). Statewide for all ages, significant increases in hospitalizations occurred but within the same causes as for the main analysis. This suggests that by restricting the definition of “heat wave” to those days with more intense heat, one may discern a greater number of significantly elevated RRs in hospitalizations, but less so among ED visits.

We minimized data suppression issues by regional aggregation of cases, yet for some of the race/ethnicity categories, low cell counts, particularly during the reference period, and underreporting combined to mask some of the relevant RR calculations.

### Preventive measures suggested

Previous studies have suggested that to reduce morbidity from some of the more severe heat-related conditions, interventions should include improving community access to air conditioning, encouraging increased fluid intake, and advising temporarily decreased physical activity (Semenza 1999). Heat-health warning systems that have been instituted in several large cities are aimed mainly at preventing heat-related mortality (Kovats and Ebi 2006). Heat wave response plans (Ebi and Schmier 2005) could be adapted with improved surveillance from studies like this one to target local physicians, emergency medical providers, and hospital staff with specific messaging about the range of health conditions that have been determined to increase locally during heat waves and suggest treatment strategies (Weir 2002; Wexler 2002). Regions far from urban centers are also home to residents of lower socioeconomic status or to socially isolated older persons (CDPH 2008), making heat wave preparedness a concern that extends beyond the city limits. Air conditioning has been shown to be among the most health-protective adaptations to heat (O’Neill 2003; O’Neill et al. 2005b). Culturally and socially appropriate messaging through public service announcements—for example, encouraging at-risk groups to access cooling centers, and ensuring availability of transportation to those centers—before a heat wave starts can save lives. Expanded education of at-risk groups and their caregivers on how to detect signs and symptoms and prevent heat-related illness is needed. Education should also emphasize the need to seek immediate medical assistance for heat-related illness, because these conditions often progress very rapidly and therefore urgently require professional medical intervention.

This study also suggests some appropriate types of acute heat-related illnesses to serve as indicators in syndromic surveillance programs that collect reports in real time from EDs or doctors’ offices to identify rapidly emerging morbidity during a heat wave (Josseran et al. 2007). In the present study, the nonspecific internal-cause grouping may have masked or minimized some heat-related health effects, suggesting that this grouping may not offer adequate sensitivity to be a good syndromic marker for heat vulnerability.

Heat waves can create community medical emergencies, and extreme heat events are likely to increase in frequency, intensity, and duration in the coming years, as stated by the Intergovernmental Panel on Climate Change (Confalonieri et al. 2007). Hospitals and EDs could adapt their procedures to meet added demands that heat waves can place on local health care systems, such as reducing elective medical services to free staff and beds, treating patients in nontraditional locations, and coping with the loss of hospital personnel who are themselves stricken by heat-related morbidities (Schull 2006). This study may help clarify some targeted interventions that clinicians could make among at-risk patient population groups when heat waves are predicted to occur locally.

## Conclusions

This work suggests that the 2006 heat wave had a substantial effect on morbidity in California and that heat-related causes showed statistically significant variation across different regions. The large RR for the Central Coast region, where there are typically moderate summer temperatures, suggests that residents are less acclimatized, have less access to climate-controlled environments, or may not consider themselves vulnerable to heat waves and therefore do not take measures to prevent heat stress. A much wider range of elevated RRs across different causes and ages was reflected in the ED visits than in the hospitalization data during the heat wave, which suggests the importance of evaluating both ED visits and hospitalizations. Besides older residents with recognized heat vulnerabilities, children showed significant elevated risk for some morbidities. Strategies to prevent heat-related illness during extreme heat events should include messages and information dissemination targeted toward parents, caregivers, and other guardians of young children, continued outreach to the elderly and especially to socially isolated individuals, and geographically targeted messages about health risks of heat exposure and heat stress. By better understanding heat wave effects on morbidity, local communities can develop appropriate public health interventions and increase their adaptive capacity to cope with heat waves when they happen—both today and in a globally warming future.

## Figures and Tables

**Table 1:** California RRs for all-cause ED visits and hospitalizations, evaluated from combined primary and secondary diagnoses during the 2006 heat wave (15 July to 1 August 2006), versus the reference period (8–14 July and 12–22 August 2006), summarized by region, age, and race/ethnicity.

	ED visits			Hospitalizations		
Category	Reference period	Heat-wave period	RR (95% CI)	Reference period	Heat-wave period	RR (95% CI)
California statewide	485,785	501,951	1.03 (1.02–1.04)	191,826	193,008	1.01 (1.00–1.01)
Geographic region						
Central Coast	84,958	89,546	1.05 (1.04–1.06)	32,514	33,064	1.02 (1.00–1.03)
Central Valley	84,445	87,555	1.04 (1.03–1.05)	31,113	31,211	1.00 (0.99–1.02)
North Central	20,330	21,131	1.04 (1.02–1.06)	6,720	6,802	1.01 (0.98–1.05)
North Coast	25,290	26,502	1.05 (1.03–1.07)	7,997	8,197	1.03 (0.99–1.06)
South Coast	209,737	215,331	1.03 (1.02–1.03)	90,552	90,752	1.00 (0.99–1.01)
Southeast Desert	61,025	61,886	1.01 (1.00–1.03)	22,930	22,982	1.00 (0.98–1.02)
Age (years)						
0–4	47,337	49,800	1.05 (1.04–1.07)	33,279	33,397	1.00 (0.99–1.02)
5–64	352,915	364,219	1.03 (1.03–1.04)	102,935	103,381	1.00 (1.00–1.01)
≥ 65	85,533	87,932	1.03 (1.02–1.04)	55,612	56,230	1.01 (1.00–1.02)
Race/ethnicity						
Asian/Pacific Islander	23,431	23,942	1.02 (1.00–1.04)	14,741	14,880	1.01 (0.99–1.03)
African American	50,439	51,404	1.02 (1.01–1.03)	15,621	15,314	0.98 (0.96–1.00)
Latino/Hispanic	147,620	153,627	1.04 (1.03–1.05)	58,630	58,893	1.00 (0.99–1.02)
Native American/Alaska Native	1,677	1,764	1.05 (0.98–1.13)	506	485	0.96 (0.85–1.09)
Other	17,327	18,123	1.05 (1.02–1.07)	5,420	5,648	1.04 (1.00–1.08)
Unreported	22,328	23,779	1.07 (1.05–1.08)	1,963	1,939	0.99 (0.93–1.05)
White	222,963	229,312	1.03 (1.02–1.03)	94,945	95,849	1.01 (1.00–1.02)

**Table 2:** Statewide ED visits and hospitalization RRs, for all ages, all race/ethnicity groups combined, during the 2006 California heat wave (15 July to 1 August 2006), versus the reference period (8–14 July and 12–22 August 2006), evaluated from combined primary and secondary diagnoses.

		ED visits			Hospitalizations		
Diagnosis	ICD-9-CM code	Reference period	Heat-wave period	RR (95% CI)	Reference period	Heat-wave period	RR (95% CI)
All causes	All	485,785	501,951	1.03 (1.02–1.04)	191,826	193,008	1.01 (1.00–1.01)
Internal causes	0–799.9	386,229	399,699	1.03 (1.03–1.04)	172,864	173,843	1.01 (1.00–1.01)
Diabetes	250	37,321	38,315	1.03 (1.01–1.04)	27,644	27,920	1.01 (0.99–1.03)
Electrolyte imbalance	276	30,076	35,020	1.16 (1.15–1.18)	25,647	28,003	1.09 (1.07–1.11)
Cardiovascular diseases	390–398, 402, 404–429, 440–448	45,613	46,515	1.02 (1.01–1.03)	48,327	48,821	1.01 (1.00–1.02)
Acute MI	410	2,822	2,869	1.02 (0.96–1.07)	3,630	3,688	1.02 (0.97–1.06)
Cerebrovascular disease	430–438	7,397	7,250	0.98 (0.95–1.01)	8,266	8,138	0.98 (0.95–1.02)
Respiratory illnesses	460–519	64,051	64,213	1.00 (0.99–1.01)	36,753	37,226	1.01 (1.00–1.03)
Nephritis and nephrotic syndrome	580–589	12,185	12,935	1.06 (1.04–1.09)	14,118	14,801	1.05 (1.02–1.07)
Acute renal failure	584	5,085	5,839	1.15 (1.11–1.19)	6,541	7,288	1.11 (1.08–1.15)
Heat-related illnesses	992	403	2,537	6.30 (5.67–7.01)	61	619	10.15 (7.79–13.43)

**Table 3:** RRs (95% CIs) for four selected causes of morbidity in California during the 2006 heat wave (15 July to 1 August 2006) versus the reference period (8–14 July and 12–22 August 2006), evaluated from combined primary and secondary diagnoses.

	Heat-related illnesses		Electrolyte imbalance		Acute renal failure		Nephritis	
Demographic	ED visits	Hospital admissions	ED visits	Hospital admissions	ED visits	Hospital admissions	ED visits	Hospital admissions
California statewide	6.30 (5.67–7.01)	10.15 (7.79–13.43)	1.16 (1.15–1.18)	1.09 (1.07–1.11)	1.15 (1.11–1.19)	1.11 (1.08–1.15)	1.06 (1.04–1.09)	1.05 (1.02–1.07)
Geographic region								
Central Coast	23.05 (15.05–37.10)	Insufficient data	1.22 (1.17–1.26)	1.15 (1.10–1.19)	1.19 (1.09–1.30)	1.14 (1.05–1.23)	1.09 (1.03–1.15)	1.05 (1.00–1.11)
Central Valley	7.35 (6.01–9.07)	17.10 (9.08–36.30)	1.23 (1.19–1.28)	1.13 (1.08–1.18)	1.27 (1.16–1.41)	1.22 (1.12–1.33)	1.15 (1.08–1.23)	1.10 (1.03–1.17)
North Central	8.56 (5.24–14.82)	Insufficient data	1.22 (1.13–1.32)	1.09 (1.00–1.18)	1.37 (1.11–1.71)	1.23 (1.02–1.49)	1.10 (0.95–1.26)	1.05 (0.92–1.21)
North Coast	5.05 (3.14–8.49)	Insufficient data	1.18 (1.10–1.26)	1.03 (0.95–1.11)	1.07 (0.88–1.29)	0.97 (0.82–1.14)	1.00 (0.95–1.26)	0.92 (0.82–1.02)
South Coast	5.05 (4.12–6.25)	6.29 (3.95–10.49)	1.11 (1.09–1.14)	1.07 (1.04–1.09)	1.10 (1.04–1.16)	1.09 (1.04–1.14)	1.04 (1.00–1.07)	1.04 (1.01–1.08)
Southeast Desert	3.36 (2.74–4.15)	4.36 (2.72–7.29)	1.16 (1.11–1.22)	1.10 (1.04–1.16)	1.10 (0.98–1.23)	1.08 (0.97–1.20)	1.04 (0.96–1.12)	1.06 (0.98–1.14)
Age (years)								
0–4	6.17 (2.58–17.88)	Insufficient data	1.19 (1.10–1.30)	1.06 (0.97–1.16)	Insufficient data	0.61 (0.31–1.15)	0.81 (0.44–1.49)	0.73 (0.49–1.10)
5–64	5.43 (4.83–6.13)	7.00 (4.90–10.28)	1.18 (1.15–1.20)	1.08 (1.05–1.11)	1.21 (1.13–1.28)	1.15 (1.09–1.22)	1.07 (1.03–1.11)	1.06 (1.02–1.10)
≥ 65	10.87 (8.39–14.31)	14.23 (9.56–22.08)	1.15 (1.12–1.18)	1.10 (1.08–1.13)	1.12 (1.07–1.17)	1.10 (1.05–1.14)	1.05 (1.02–1.09)	1.04 (1.01–1.07)
Race/ethnicity								
Asian/Pacific Islander	11.38 (5.53–27.14)	Insufficient data	1.14 (1.07–1.20)	1.09 (1.02–1.15)	1.09 (0.95–1.25)	1.08 (0.96–1.21)	1.06 (0.98–1.16)	1.03 (0.95–1.11)
African American	5.29 (3.83–7.45)	6.57 (2.95–17.25)	1.17 (1.11–1.23)	1.08 (1.02–1.14)	1.16 (1.05–1.30)	1.13 (1.03–1.25)	1.05 (0.99–1.12)	1.07 (1.00–1.14)
Latino/Hispanic	6.50 (5.30–8.02)	10.85 (6.14–20.87)	1.16 (1.12–1.20)	1.06 (1.02–1.10)	1.15 (1.05–1.25)	1.10 (1.02–1.18)	1.05 (1.00–1.11)	1.05 (1.00–1.10)
Native American/Alaska Native	Insufficient data	Insufficient data	1.09 (0.82–1.45)	0.97 (0.68–1.38)	0.77 (0.30–1.90)	0.71 (0.28–1.73)	1.59 (0.98–2.62)	0.97 (0.59–1.60)
Other	7.00 (3.70–14.60)	Insufficient data	1.20 (1.10–1.32)	1.07 (0.96–1.20)	1.06 (0.82–1.38)	1.03 (0.82–1.30)	0.94 (0.80–1.11)	1.02 (0.87–1.19)
Unreported	5.06 (3.03–8.91)	Insufficient data	1.24 (1.11–1.38)	1.15 (0.95–1.40)	1.18 (0.75–1.84)	0.92 (0.62–1.34)	1.17 (0.92–1.50)	0.94 (0.71–1.25)
White	6.28 (5.43–7.30)	10.06 (7.13–14.59)	1.16 (1.14–1.19)	1.11 (1.08–1.13)	1.16 (1.10–1.22)	1.13 (1.08–1.18)	1.07 (1.03–1.11)	1.05 (1.02–1.08)
